# Phytomediated Photo-Induced Green Synthesis of Silver Nanoparticles Using *Matricaria chamomilla* L. and Its Catalytic Activity against Rhodamine B

**DOI:** 10.3390/biom10121604

**Published:** 2020-11-26

**Authors:** Abdulmohsen Ali Alshehri, Maqsood Ahmad Malik

**Affiliations:** Chemistry Department, Faculty of Sciences, King Abdulaziz University, P.O. Box 80203, Jeddah 21589, Saudi Arabia; aayalshehri@kau.edu.sa

**Keywords:** biomolecules, *Matricaria chamomilla*, phytochemicals, photo-induced, catalytic activity

## Abstract

The bio-fabrication of silver nanoparticles (AgNPs) was carried out through the facile green route, using the aqueous extract of *Matricaria chamomilla* L. Herein, we have developed a cost-efficient, ecofriendly, and photo-induced method for the biomolecule-assisted synthesis of AgNPs using an aqueous extract of *Matricaria chamomilla* L. as a bio-reducing and capping/stabilizing agent. The biomolecule-capped AgNPs were confirmed from the surface plasmon resonance (SPR) band at λ_max_ = 450 nm using a UV–visible spectrometer. The stability of the AgNPs was confirmed by recording the UV–visible spectra for a more extended period, and no precipitation was observed in the sol. The morphology and structure of photo-induced biomolecule-capped AgNPs were characterized by different microscopic and spectroscopy techniques such as TEM, SEM, EDX, XRD, and FTIR analysis. The role of phytochemicals as reducing and stabilizing agents was confirmed by comparative FTIR analysis of the AgNPs and pure *Matricaria chamomilla* L. aqueous extract. The obtained result shows that the AgNPs are mostly spherical morphology with an average size of about 26 nm. Furthermore, the thermal stability of biomolecule-capped AgNPs was examined by TGA-DTG analysis that showed a weight loss of approximately 36.63% up to 800 °C. Moreover, the potential photocatalytic activity of photo-induced AgNPs against Rhodamine B (RB) was examined in the presence of UV light irradiation. The catalyst reusability, the effect of catalyst dosage and initial dye concentration, and the effect of the temperature and pH of the reaction medium were also assessed.

## 1. Introduction

Nanomaterials with improved features depending on their size, shape, and structure are at the leading edge of nanotechnology’s swiftly emerging field. Their unique size and shape make them excellent and very important in numerous human activity fields like catalysis, drug delivery, bioimaging and photothermal therapy, water purification, and environmental waste treatment [1,2,3,4,5,6]. Various physical and chemical methods including hydrothermal [7], microwave [8], sonochemical [9], electrochemical [10], photochemical [11], radiation-assisted [12], and chemical reduction methods [13,14] have been employed for the synthesis of silver nanoparticles. These methods need high energy, high pressure, high operation cost, and toxic reagents as external reducing and capping or stabilizing agents. Therefore, green synthesis of nanoparticles using biological resources such as plants, bacteria, algae, and fungi has gained interest from researchers [15,16,17,18]. In recent times, various plant sources have been used to prepare stable silver nanoparticles by avoiding hazardous reagents as external reducing and capping agents [19,20,21], thus providing an alternative synthetic technique for the preparation of silver nanoparticles compared to conventional physical and chemical routes. The phytochemical-mediated synthetic approach is a clean, effective, non-toxic, and nature-friendly viable approach for silver nanoparticles (AgNPs) synthesis to combat the problems associated with physical and chemical routes [3,4,5]. Nowadays, mostly the plant extract as a primary source of biomass after various advantages such as simple, economical in nature, user-friendly, easy to eliminate, and establishment with aseptic conditions and maintenance is used compared to other biological systems. Plant extracts act as a capping agent and cause metal nanoparticle aggregation in their respective salt solutions. Various researchers used the extracts obtained from different plant parts as a reducing agent in the synthesis of AgNPs. These plant reductants are phytochemicals, including phenol derivatives, terpenoids, flavonoids, and some plant enzymes such as reductases, hydrogenases, quinones, and their derivatives [6,15].

The importance of AgNPs among metal nanoparticles is well known due to their biological properties. It has been reported that AgNPs are proven to accelerate the potent inhibitor response against microorganisms, act as free radicle scavengers, and possess anti-inflammatory properties, having properties of antiviral, anti-angiogenetic effects, anti-bacterial, and wound healing [22,23,24]. Moreover, the photo-induced phytomediated synthesis of AgNPs from plant extract in the presence of visible light was proven a more economical and environmentally friendly method [25,26]. The literature emphasizes on the phytomediated synthesis and photo-induced route of AgNPs. The photo-induced synthesis of AgNPs has been reported from *Cynodon dactylon* and *Andrachnea chordifolia* in aqueous and ethanol extract, respectively [27,28]. The green approach for the biomolecule-mediated synthesis of AgNPs in the presence of sunlight has also been explored from extracts of *Desmostachya bipinnata*, *Xanthium strumarium*, *Polyalthia longifolia*, and *Dunaliella salina*, mostly acting as stabilizing and capping agents [29,30,31,32]. Chamomile (*Matricaria chamomilla* L.) belongs to the family Asteraceae (Compositae) and has a long history of being used as a medicinal herb throughout the world [33]. The herb, due to its therapeutic purpose, dates back to ancient Greece and Rome, where the herb was referred to by Asclepius, Galen, and Hippocrates [34]. The plant chemical constituents had proven diverse pharmacological actions and were used as anti-inflammatory, antiviral, sedative, antimicrobial, antioxidant, antispasmodic, and antiseptic agents. The potential chemical compositions with pharmacological response include blue essential oil with cosmetic significance, spiroethers, terpenoids, coumarins, and flavonoids with varied biological reactions [35,36]. In recent times, apigenin, apigenin-7-O-glucoside, quercetin, and luteolin in Matricaria chamomilla, also known as German chamomilla extract, have been determined [37,38]. Apigenin (4′,5,7-trihydroxyflavone) is a flavone under the category of natural flavonoids that is abundantly present in Matricaria chamomilla. It possesses antioxidant, anticancer, and anti-inflammatory properties [39,40]. The combination of rapid purification, liquid chromatography-mass spectrometry (LC/MS), LC/MS/MS, and nuclear magnetic resonance (NMR) was used to classify apigenin 7-O-glucoside and various acylated derivatives of apigenin-7-O-glucoside in chamomile petals [41]. Apigenin 7-O-glucoside is one of the major flavonoids present in the white florets of Matricaria chamomile with advanced anti-inflammatory, antioxidant, and anticancer properties [42,43]. Quercetin is also one of the copious flavonoids present in *Matricaria chamomilla* extract with strong anti-proliferative, antioxidant, and anti-inflammatory properties. Quercetin exhibits a wide variety of anticancer activities, and cancer-preventive properties are also indicated by accumulating data [44]. Luteolin (3,4,5,7-tetrahydroxy flavone) is a yellow crystalline flavonoid found in different plants including medicinal herbs (*Matricaria chamomile*) with antimicrobial, antioxidant, anticancer, anti-inflammatory, and neuroprotective activities [45,46]. Due to their quick, ecological, non-pathogenic, inexpensive method, the use of plants to produce AgNPs is of great interest [47]. Plant-mediated synthesis of AgNPs allows for advances in chemical and physical processes, which can be easily expanded for large-scale synthesis [48].

Our study aimed to modify and propose an alternate new synthetic method of developing silver nanoparticles with improved photocatalytic and biological responses. The process of synthesis was optimized, and various characterization techniques confirmed the developed AgNPs. The reduction reaction conditions such as temperature, pH, and extract/silver nitrate ratio were examined and optimized for a better and higher yield of AgNPs. Moreover, the photocatalytic activity of biomolecule-assisted AgNPs was examined, and efforts were made to understand the mechanism behind the synthesis of AgNPs. 

## 2. Experimental

### 2.1. Material and Methods

The analytic-grade silver nitrate (AgNO_3_ (99.0%), Molecular Weight: 169.87), Rhodamine B (C_28_H_31_ClN_2_O_3_ (95%), Molecular Weight: 479.01), Methanol CH_3_OH (99.9%), Sodium chloride (NaCl (99.0%), Molecular Weight: 58.44), and Sodium bromide (NaBr (99.0%), Molecular Weight: 102.89) were purchased from Sigma Aldrich and used without any additional purification. All the stock solutions were prepared with deionized water, and the silver nitrate solution was kept in the dark to avoid photochemical oxidation. The fresh herb *Matricaria chamomilla* L. was collected from the local market in Jeddah.

#### 2.1.1. Preparation of Plant Extract

The aqueous extract of *Matricaria chamomilla* L. was obtained after washing the herb multiple times with double-distilled water to remove all dust particles and other impurities from the surface of the plant. The leaves were air-dried under shade to eradicate the moisture. Afterward, the herb was crushed manually using mortar and pestle into a fine powder and transferred to an Erlenmeyer flask containing 250 mL of double-distilled water. The mixture was warmed up to 50 °C under constant stirring for 30 min and left for cooling for 1 hour at room temperature and then filtered over Whatman filter paper No. 1 and the obtained *Matricaria chamomilla* L. extract was stored in the dark at 4 °C for further use as a reducing and stabilizing/capping agent for the synthesis of AgNPs under sunlight. 

#### 2.1.2. Biomolecule-Assisted Synthesis of AgNPs

The photo-induced *Matricaria chamomilla* mediated AgNPs were obtained after reducing 30 mL AgNO_3_ with 30mL of *Matricaria chamomilla* L. aqueous extract under sunlight with constant stirring for 5 min. The process of Ag^+^ ion reduction under sunlight was observed from the color change from yellow to deep brown color within 10 min. The yellow color of Ag^+^ and *Matricaria chamomilla* L. extract to deep brown color conforms to the bio-reduction and stabilization/capping of AgNPs. However, the reaction mixture kept in the dark neither attained the same coloration nor produced the sharp SPR band within the given period. The reaction’s complete progress was examined by recording the sol’s UV–visible spectra in the wavelength range of 200–800 nm. The SPR band appearance within 5 min of the reaction time conforms to the fabrication of stable AgNPs. The reaction optimization was carried out by varying the extract concentration from 1% to 4% and keeping the silver nitrate concentration and irradiation time constant. 

Similarly, Ag^+^ ion concentration was varied from 0.5 to 2 mM and other variables were kept unchanged. Under optimized reaction conditions, the AgNPs sol was centrifuged at a speed of 15,000 rpm for about 30 min. The acquired precipitate was re-dispersed in deionized water and centrifuged again to eliminate all impurities and water-soluble biological residues available on the surface of the AgNPs. Finally, the biomolecule-capped AgNPs were washed several times with double-distilled water followed by methanol and dried at room temperature for further analysis and applications.

### 2.2. Characterization of AgNPs

The biomolecule-capped AgNPs from *Matricaria chamomilla* L. extract under sunlight were characterized by different spectroscopic techniques to analyze their structural and morphological properties. The initial progress and the optical properties of the AgNPs formation were investigated by UV–visible spectroscopy using a Thermo Scientific Evolution 600 UV-Vis spectrophotometer (Paisley PA4 9RF, UK) with 1cm quartz cuvettes in the range of 200–800 nm wavelength. The possible role of phytochemicals/biomolecules involved in the bio-reduction and their involvement as stabilizing/capping agents in the formation of AgNPs were observed by Fourier transform infrared (FTIR) spectroscopic analysis in the range of 4000–400 cm^−1^ using a Bruker FTIR spectrophotometer (Model: ALPHA II, Bruker, Billerica, MA, USA). The diffraction intensities of photo-induced AgNPs were recorded on a powder X-ray diffractometer (XRD) (Bruker, Karlsruhe, Germany) at an operating voltage of 40kV and a current of 30 mA supplied with CuKα radiation (λ = 1.5405 Å) in the 2θ range of 20°–80° to determine the crystal structure and the size of AgNPs by the Scherrer equation (Equation (1)):

(1) $$d=\frac{k\;\lambda }{\beta cos\theta }$$

where *d* symbolizes the crystal size of the AgNPs, *β* signifies the full width at half-length maximum (FWHM) in radians, *λ* denotes the X-ray wavelength (1.4506 Å), *θ* is the Bragg diffraction angle, and *k* is a constant (0.9). The particle size and morphology of the as-prepared AgNPs were analyzed by transmission electron microscopic (TEM) images obtained from JEOL (model JEM-2010F, Tokyo, Japan, operating at 120kV). The AgNPs in the colloidal form were dropped onto the carbon-coated copper grid and then air-dried for 2h before introducing them to the TEM. The as-prepared biomolecule-assisted AgNPs were characterized using a scanning electron microscope (SEM, S-3500N, Hitachi Co., Tokyo, Japan) fitted with an energy dispersive X-ray analysis (EDX) detector for surface morphology and their elemental composition. The AgNP micrographic images were captured at 4000×, and for elemental composition analysis, the corresponding energy-dispersive X-ray (EDX) spectra were recorded in standard and point-and-shoot modes. The thermal stability of as-prepared AgNPs was determined under a N_2_ atmosphere with a heating rate of 10 °C /min using a thermogravimetric analyzer (TGA) (Perkin-Elmer Pyris Diamond (Perkin-Elmer, Waltham, MA, USA) in the heating range of 30–800 °C.

### 2.3. Photocatalytic Activity

The catalytic efficiency of photo-induced phytochemical-capped AgNPs was evaluated for the catalytic degradation of Rhodamine B (RB) under UV irradiation. The RB dye’s stock solution was prepared by dissolving an appropriate amount in double-distilled water to achieve 1.0 × 10^−5^ M concentration of aqueous dye solution. The as-synthesized AgNPs were used as catalysts in a concentration range of 5 to 25 mg/mL. Different RB dye concentrations were also investigated to achieve optimum experimental conditions for efficient catalytic performance of AgNPs. In a typical degradation experiment, 50 mL of 1.0 × 10^−5^ M dyes solution was taken in a three-neck flask, and 50 mg/mL was added to the RB dyes solutions under continuous stirring under dark conditions to maintain the adsorption–desorption equilibrium. The catalytic degradation of the above solution was carried out in a UV chamber having a UV light source of 11 W. After every 10 min of reaction time, reaction samples were collected from the UV chamber, centrifuged for 5 min at 10,000 rpm, and the progress of the degradation reaction was monitored by recording the absorption using a UV–visible spectrophotometer. A control set of degradation experiments was carried out in the absence of AgNPs, and the absorbance was recorded periodically. The following equations, respectively, measured the percent degradation and first-order rate constant (*k*) for RB dye decolorization under UV light irradiation (Equations (2) and (3)).

(2) $$Photocatalytic\;efficiency\;\left(\%\right)=\frac{C_{o}-C_{t}}{C_{o}}\;\times 100=\frac{A_{o}-A_{t}}{A_{o}}\times 100$$

(3) $$\ln\left(\frac{A_{o}}{A_{t}}\right)=kt$$

where *C_o_* and *C_t_* are the initial concentration and final concentration after the reaction time (*t*) of Rhodamine B, respectively. Additionally, *A_o_* is the initial absorbance, and *A_t_* is the absorbance of the dye at the reaction time *t*.

## 3. Results and Discussion

### 3.1. UV–Visible Spectroscopy

**Figure 1:**
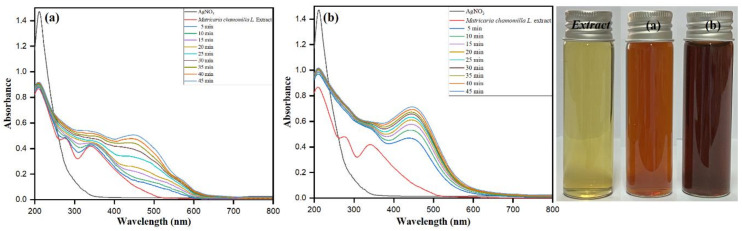
UV–vis absorption spectra of biomolecule-capped AgNPs recorded at 5 min time intervals for 5 to 45 mins (**a**) in bright sunlight ([AgNO_3_ = 2.0 mM, [*Matricaria chamomilla* L.] = 4%) and (**b**) in dark reaction conditions ([AgNO_3_ = 2.0 mM, [*Matricaria chamomilla* L.] = 4%). (Optical images of *Matricaria chamomilla L. extract*: AgNPs under dark conditions (**a**), and AgNPs under sunlight (**b**)).

The extracellular synthesis of metallic nanoparticles is due to several metabolites and reductive biomolecules in the plant extract, responsible for reducing metal ions into nanoparticles and stabilizing/capping them to protect them from self-aggregation and controlling the size and growth process. In the present study, an aqueous extract of *Matricaria chamomilla* L. was used as a bio-reducing and stabilizing/capping agent for the bio-fabrication of AgNPs. After inoculated in an aqueous solution of AgNO_3_ in the presence of bright sunlight, the Matricaria chamomilla L. extract attained an instant change in color within a few minutes, while it failed to attain the same degree of color change in dark conditions. The prime visual inference of color change is after the synthesis of AgNPs, which was observed from the sharp SPR band at 450 nm after 5 min exposure of sunlight, as depicted in Figure 1a. Meanwhile, at the 45 min time lag, sharp and steep intense peaks emphasize the potent biomolecule-assisted synthesis of AgNPs in the presence of bright sunlight compared to dark conditions. The sharp surface plasma resonance (SPR) intense band at 450 nm is formed behind the oscillation of the conduction electrons [49]. The exciting free electrons in AgNPs, while absorbing visible light, are responsible for the presence of an intense SPR band [50]. 

The eminent UV–visible absorption spectroscopy, being a novel technique, is involved in determining the biomolecule-assisted synthesis of AgNPs. The sample from the extract reaction mixture was withdrawn at regular time intervals under dark conditions and in the presence of bright sunlight and screened between 200 and 800 nm through UV–visible spectroscopy (Figure 1a,b). It is also evident from the optical images shown in Figure 1 that the color transition of the silver sol from light brown under dark conditions to dark brown under bright sunlight takes place, which conforms to the formation of stable AgNPs under optimum reaction conditions ([*Matricaria chamomilla* L.] = 4%, [AgNO_3_] = 2mM, bright sunlight exposure for 45 min). The exposure of the extract mixture solution sample after 45 min bright sunlight exposure showed the sharp, intense SPR band at 450 nm corresponding to the characteristic surface plasmon resonance of AgNPs having λ_max_ values at the range between 400 and 500 nm [51]. In dark conditions, after running the sample extract solution for 45 min, the steep SRP band between 400 and 500 nm showed an insufficient growth of AgNPs. The shape, nature, surrounding media, and size of the developed nanoparticles emphasize the presence of single or multiple SPR bands [52,53]. In our study, the observance of an intense sharp SPR band at 450 nm favors the growth of spherical AgNPs, where the presence of other SPR bands implicates possible variation in the shape of the AgNPs. The present study’s reaction mixture showed multiple SPR bands indicating both spherical and some irregular AgNPs, which was supported by the obtained results of SEM and TEM images. 

### 3.2. AgNO_3_ Concentration Optimization 

**Figure 2:**
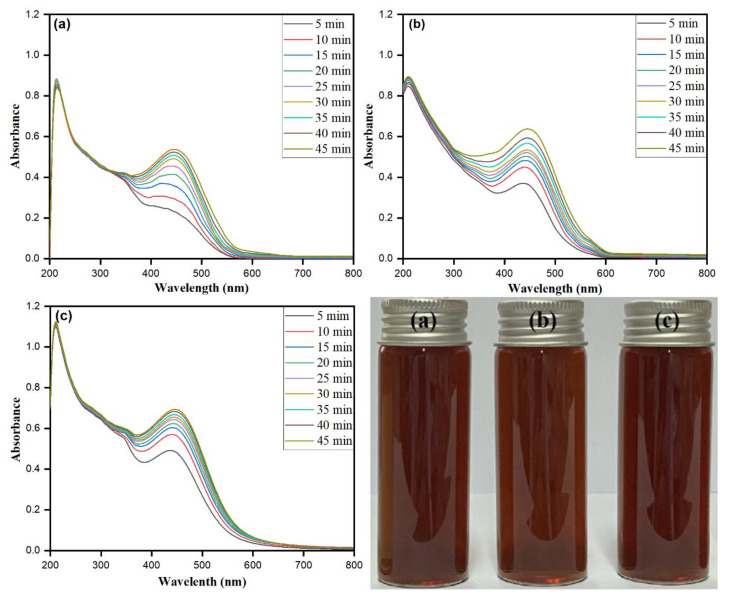
UV–visible absorption spectra recorded as a function of different AgNO_3_ concentrations (0.5 to 2 mM) for biomolecule-assisted synthesis of AgNPs: (**a**) 0.5, (**b**) 1.0, and (**c**) 1.5 mM, at constant *Matricaria chamomilla L*. extract (3.0%) and sunlight exposure for 5 to 45 min. (Optical images of AgNPs at different AgNO_3_ concentrations: (**a**) 0.5, (**b**) 1.0, and (**c**) 1.5 mM, and constant *Matricaria chamomilla L*. extract (4.0%)).

The standard ratio of *Matricaria chamomilla* L. extract with the silver ions of the reaction mixture at a particular inoculum dose has an impact on the optimization and controlled synthesis of stable AgNPs. The reaction process needs to be optimized using varying concentrations of AgNO_3_ from 0.5 to 2 mM at 4% *Matricaria chamomilla* L. extract inoculum dose and 45 min bright sunlight exposure. The different concentrations of AgNO_3_, i.e., 0.5 mM at 449 nm, 1.0 mM at 450 nm, and 1.5 mM at 450 nm at various time intervals, are shown in Figure 2a–c. The observed dark color of the reaction mixture at varying AgNO_3_ concentrations with increased sunlight exposure from 5 to 45 min accentuates that the AgNO_3_ concentration directly influences the particle size distribution [54,55]. The increase in the concentration of AgNO_3_ leads to a rise in the biomolecule-assisted synthesis of AgNPs, which can be clearly observed in Figure 2a–c. After the addition of the 4% *Matricaria chamomilla* L. extract to different AgNO_3_ concentrations, the color intensity of the reaction mixture changes to stable dark brown on increasing the AgNO_3_ concentration, as shown in Figure 2 (optical images). The intensity and the sharpness of the SPR band at varying concentrations increase to 2 mM at each time interval and then show steep broadening of the bands. The simultaneous increase in time and the concentration of AgNO_3_ leads to shifting the SPR band towards the higher frequency with a redshift. The proceeding time intervals from 5 to 45 min with varying concentrations, i.e., 0.5, 1.0, and 1.5 mM, showed a redshift from 445 to 449 nm, 446 to 450 nm, and 447 to 450 nm, respectively. Thus, the increase in the concentration of AgNO_3_ from 0.5 to 1.5 mM after 45 min bright sunlight exposure showed a redshift with the SPR band nearly constant at 450 nm. The pure 2 mM AgNO_3_ solution did not show any peak in the wavelength range of 300–800 nm (Figure 1a). Meanwhile, in our study, 2.0 mM AgNO_3_ concentration at 4% *Matricaria chamomilla L* extract at 45 min bright sunlight exposure time was optimized as the parameters for the small particle size and the controlled biomolecule-assisted synthesis of AgNPs.

The stability of as-prepared AgNPs was determined by keeping the silver sols at 30 °C for several days. During this time, we did not observe any kind of precipitation or turbidity in the reaction mixture. The nature of reducing agents and capping or stabilizing agents present in the reaction mixture specifies the optical properties of the AgNPs. To confirm the complete conversion of Ag^+^ to Ag^0^, NaCl and NaBr were added to the silver sole at the same concentration as AgNO_3_ to ensure the complete conversion of Ag^+^ to Ag^0^. It was clearly observed that no white precipitation of Ag^+^ as AgCl and yellowish precipitation of Ag^+^ as AgBr took place, which confirms the complete reduction/conversion of Ag^+^ to Ag^0^. Therefore, the amount of AgNO_3_ used in the preparation of AgNPs approximately represented the amount of AgNPs produced.

On the other hand, the concentration of biomolecule-capped AgNPs was calculated by the method reported by Al-Ghamdi et al. [22]. The average number of atoms per nanoparticle was determined by the following equation (Equation (4)):

(4) $$N=\frac{\pi \rho D^{3}}{6M}N_{A}$$

where *N* = number of atoms per nanoparticle, *π* = 3.14, *ρ* = density of face-centered cubic silver = 10.5 g/cm^3^, *D* = average diameter of nanoparticles = 26 nm = 26 × 10^−7^ cm, *M* = atomic mass of silver = 107.868 g, and *N_A_* = number of atoms per mole (Avogadro’s number = 6.023 × 10^23^ mol^−1^). Therefore, we presumed 100% reduction of all silver ions (Ag^+^) to silver nanoparticles (Ag^0^). The *N* = 539,272.1315 (average number of atoms per nanoparticle) and nanoparticle solution molar concentration *C* were determined by Equation (5) and Equation (6), respectively.

(5) $$N=\;\frac{\left(\pi \;\times \;10.5\;\times \;\left(26.0\;\times \;10^{-7}\right)³\;\times \;6.023\;\times \;10^{23}\right)}{6\;\times \;107.868}$$

(6) $$C=\frac{N_{Total}}{NVN_{A}}$$

where *C* = nanoparticle solution molar concentration, *N_Total_* = total amount of silver atoms added in the form of AgNO_3_ = 2.0 mM = 0.002 M, *N* = 539,272.1315 (number of nanoparticle’s atoms), *V* = 60 ml = 0.06 L (volume of reaction solution in L), and *N_A_* = Avogadro (6.023 = 10^23^). The following equation calculated the molar concentration of as-prepared AgNPs, *C* = 6.18 × 10^−8^ mol/L (Equation (7)).

(7) $$C\;=\;\frac{\left[0.002\;\times \;6.023\;\times \;10^{23}\right]}{539272.1315\;\times \;0.06\;\times \;6.023\;\times \;10^{23}}$$

### 3.3. Matricaria chamomilla L. Extract Inoculum Dose

**Figure 3:**
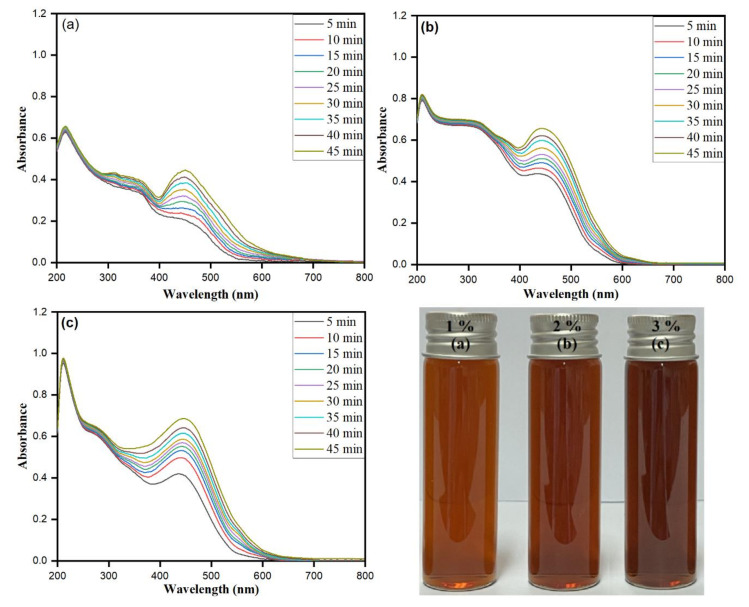
UV–visible absorption spectra of AgNPs recorded as a function of *Matricaria chamomilla* L. extract inoculum dose from 1% to 4%: (**a**) 1%, (**b**) 2.0%, and (**c**) 3%, at constant AgNO_3_ concentration (2 mM) and exposure for 5 to 45 min to bright sunlight. (Optical images of AgNPs at different *Matricaria chamomilla* L. extract concentrations: (**a**) 1%, (**b**) 2.0%, and (**c**) 3%, and constant AgNO_3_ concentration (2 mM)).

The inoculum *Matricaria chamomilla L* extract dose (*v*/*v*) directly affects the biomolecule-assisted synthesis of AgNPs. The optimization was carried for the reducing agent in the reaction mixture via keeping the other parameters constant and varying the *Matricaria chamomilla* L. extract inoculum dose (*v*/*v*) from 1.0% to 4.0% at 45 min sunlight exposure and 2 mM AgNO_3_ concentration. Figure 3a–c accentuate an increase in the SRP band pattern with the intensity of the changing color upon increased inoculum dose at exposure time intervals between 5 and 45 min. The reaction mixture’s color was observed to darken upon each *Matricaria chamomilla* L. extract inoculum dosage at increased screening time intervals. The biomolecule-assisted synthesis of AgNPs increases with an increase in each inoculum dosage as observed from the darkening response of the reaction mixture with prominent and sharp SPR bands with increased time intervals. The presence of sharp SPR bands nearly at 450 nm for *Matricaria chamomilla* L. extract inoculum dosages of 1%, 2%, and 3% directs the synthesis of irregular shapes along spherical isotropic AgNPs, which was further confirmed by SEM and TEM images. The close perusal of Figure 3 for the *Matricaria chamomilla* L. extract inoculum dose of 1% showed broader SPR bands with less intensity, emphasizing the synthesis of different shaped and fewer AgNPs in large numbers.

The further increase in *Matricaria chamomilla L* extract inoculum dosage by 2.0% showed the SPR band nearly at 450 nm with increased intensity of the shoulder SPR band at lower wavelengths. The synthesis of anisotropic AgNPs further increased with the increase in the percentage of *Matricaria chamomilla* L. extract inoculum dose, which was observed from the subsequent broadening of main SPR bands with the shifting of shoulder peaks towards lower wavelengths. The study reflects that, while changing the volume of *Matricaria chamomilla* L. extract, variation λ_max_ is observed, signifying the possibilities of the obtained different particle size AgNPs [56]. In Figure 3c, the obtained projecting intensity SPR band at 450 nm further conforms to the synthesis of spherical isotropic AgNPs in large numbers when comparing to 1.0% and 2.0% *Matricaria chamomilla* L. extract inoculum dosages. However, the screening of absorption bands of *Matricaria chamomilla* L. extract inoculum dosages of 1.0%, 2.0%, and 3.0% showed a redshift from 443 to 449 nm, 445 to 450 nm, and 447 to 450 nm, respectively. The redshift specifies the obtained increased size of AgNPs [57]. Thus, the relatively higher 4.0% *Matricaria chamomilla* L. extract inoculum dosage was approximated as the optimum dose for attaining the synthesis of stable AgNPs. 

### 3.4. The Mechanism behind the Biomolecule-Assisted Synthesis of AgNPs

**Figure 4:**
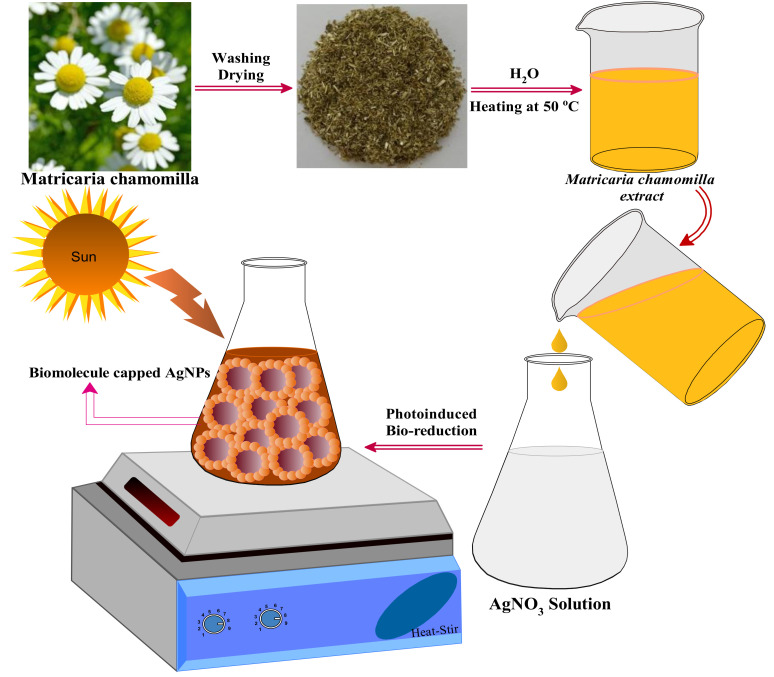
Biomolecule-assisted synthesis of capped AgNPs upon the photo-induced chemical reduction synthetic process.

The current study was related to the photo-induced biomolecule-assisted synthesis of AgNPs’ initiation upon the absorption of bright sunlight within 5–45 min exposure in the presence of photosensitive molecules of *Matricaria chamomilla* L extract. A schematic representation of the biomolecule-assisted synthesis of AgNPs under photo-induced experimental conditions is shown in Figure 4. The photosensitive chemical composition of *Matricaria chamomilla* L. extract included terpenoids, coumarins, flavonoids, spiroethers, proteins, and sugars [30]. Terpenoids, flavonoids, and sugars are effective reducing agents, whereas proteins and glucose molecules with other phytochemicals play the role of capping/stabilizing agents in the synthesis of AgNPs [58].

**Figure 5:**
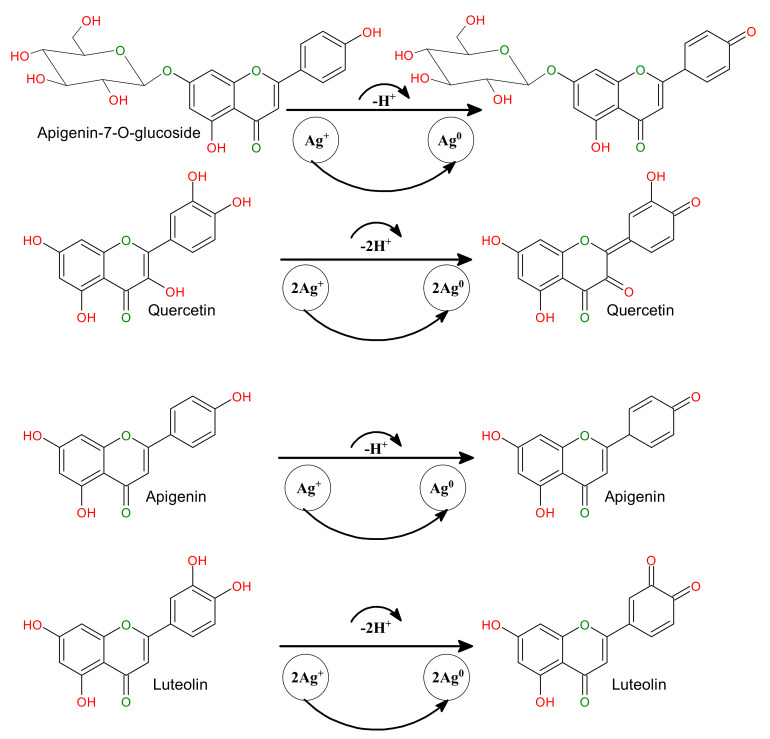
A plausible mechanism for the green synthesis of biomolecule-capped AgNPs.

The proposed mechanisms behind the biomolecule-assisted synthesis of AgNPs using *Matricaria chamomilla* L. extracts as bio-reducing and capping/stabilizing agents have been explored on the bases of FTIR analysis of the *Matricaria chamomilla* L. aqueous extract, the photocatalytic effect of sunlight irradiation to accelerate the photoreaction, and the presence of the phytochemicals present in the *Matricaria chamomilla* L. extract. The observed peaks in the FTIR spectra clarify the possible contribution of –OH groups in the synthesis and stabilization of AgNPs. The photo-induced biomolecule-assisted synthesis of AgNPs can be understood from the resonance structures of flavonoids. The exposed flavonoids to bright sunlight absorb photons of energy and release electrons after excitation, and the deboning of –OH bonds reduces Ag^+^ to Ag^0^. The presence of protein molecules in *Matricaria chamomilla* L. extract is believed after the capping and stabilization of AgNPs. The enol form of the flavonoid gets converted to the stable keto form, as shown by the resonant structures in the systematic Figure 5. The release of H^+^ ions in the reaction mixture after bright sunlight irradiation reduces Ag^+^ to Ag^0^ while absorbing photons that converted the phenol groups to the stable keto form in flavonoids apigenin-7-*O*-glucoside and apigenin. Similarly, the two Ag^+^ were reduced to Ag^0^ in flavonoids quercetin and luteolin in the presence of bright sunlight and upon the absorption of two photons and the release of two H^+^ cations, as explored in the systematic Figure 5. 

**Figure 6:**
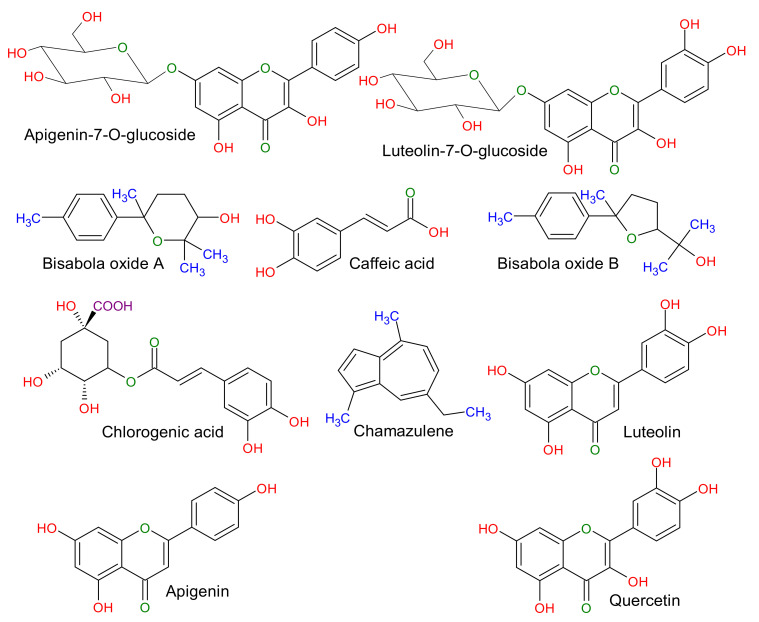
Various chemical constituents of a herb extract (*Matricaria chamomilla* L.).

Many metabolites and reductive biomolecules in the plant extract are responsible for reducing metal ions and stabilizing/capping nanoparticles. These include terpenoids, flavones, ketones, aldehydes, amides, carboxylic acids, proteins, vitamins, and carbohydrates [59]. *Matricaria chamomilla* is considered among the influential group of cultivated medicinal plants, which comprises a large group of therapeutically interesting and active compound classes. The most common chamomile constituents are sesquiterpenes, flavonoids, coumarins, and polyacetylenes (Figure 6) [60]. Chamomile extract contains the most bioactive phenolic compounds, such as apigenin, apigenin-7-O-glucoside, luteolin and luteolin-7-O-glucoside (flavone), quercetin and rutin (flavonols), herniarin and umbelliferone (coumarin), chlorogenic and caffeic acid (phenylpropanoids), and naringenin (flavanone) [61].

**Figure 7:**
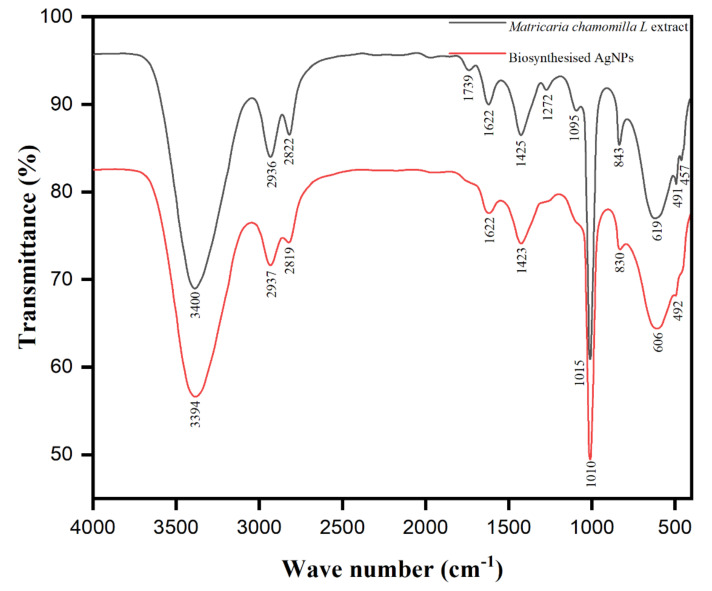
FTIR spectra of the *Matricaria chamomilla* L. extract and biomolecule-capped AgNPs.

The presence of chamomile’s various chemical constituents (*Matricaria chamomilla* L.) is shown in the systematic Figure 6. To identify the functional group involved in reducing Ag+ to Ag0 and capping or stabilizing the as-synthesized biomolecule-assisted AgNPs, Fourier transform infrared spectroscopy (FTIR) is an essential tool for this observation. FTIR spectroscopy was used to classify the major functional groups present on the surface of nanoparticles synthesized by Matricaria chamomilla extract, responsible for reducing silver ions (Ag^+^) to silver nanoparticles (Ag^0^). The FTIR analysis of *Matricaria chamomilla* L. extract was carried out to comprehend different functional groups’ involvement after reducing and stabilizing AgNPs, as represented in Figure 7. The FTIR of both the control as powdered AgNPs and the *Matricaria chamomilla* L. extract has similar peaks, emphasizing the biomolecule-assisted synthesis of AgNPs. The peak at 3400 cm^-1^ was attained due to the –OH group of polyphenolic compounds, as highlighted in the proposed mechanism of changing the enol form into quinonoid after reducing flavonoids (Figure 5). The observed bands at 1739 and 1622 cm^−1^ revealed protein molecules in *Matricaria chamomilla* L. extract and are responsible for stabilizing AgNPs. The peak at 1452 cm^−1^ may be ascribed to the methylene scissoring vibration in protein molecules. The protein carbonyl groups possess a strong binding ability and are thus expected to have strong capping and anti-agglomeration properties in the biomolecule-assisted synthesis of AgNPs. The appearance of peaks at 1222 and 1095 cm^−1^ accentuates the presence of phenolic (–OH) groups of terpenoids and flavonoids primarily responsible for the biomolecule-assisted synthesis of AgNPs in *Matricaria chamomilla* L. extract. Besides, the sharp peak at 1015 cm^−1^ was observed due to C-O-C stretch after the reduction of Ag^+^ ions in *Matricaria chamomilla* L. extract. The *Matricaria chamomilla* L. extract, as such, possesses phytochemicals, including flavonoids, sesquiterpenes, polyacetylenes, and coumarins. The presence of phenolic compounds in plant extract has been reported after the reduction of Ag^+^ ion to AgNPs in the presence of bright sunlight [62]. Our observed results from the FTIR analysis emphasize that various functional groups of phytochemicals in *Matricaria chamomilla* L. extract possess higher stability in the biomolecule-assisted synthesis of AgNPs.

### 3.5. Morphological Characterization of Biomolecule-Capped AgNPs

**Figure 8:**
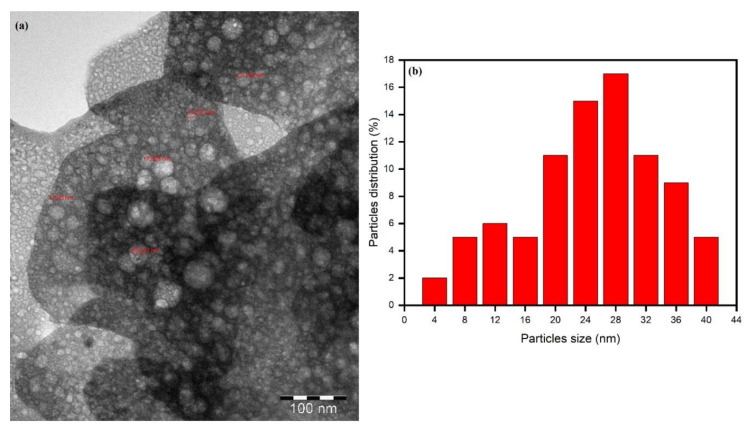
TEM image of optimized AgNPs (**a**) and AgNPs histogram showing the size distribution (**b**).

Transmission electron microscopy (TEM) is a useful nanoscience technique and nanotechnology to investigate the surface morphology and size of as-synthesized nanoparticles. The TEM images of as-prepared AgNPs using phytochemicals present in *M. chamomilla* extract as bio-reducing and capping/stabilizing agents are depicted in Figure 8a. The TEM image shows the abundance of approximately spherical-shaped biomolecule-capped AgNPs with the size ranging from 5 to 40 nm. The TEM analysis revealed that the AgNPs are efficiently stabilized by phytochemicals present in *Matricaria chamomilla* L. aqueous extract. A TEM image size distribution histogram shows that AgNPs range from 5 to 40 nm in size with an average size of 26 nm, as shown in Figure 8b. The size distribution histogram shows that the maximum AgNPs were in the range of 5 to 15 nm.

**Figure 9:**
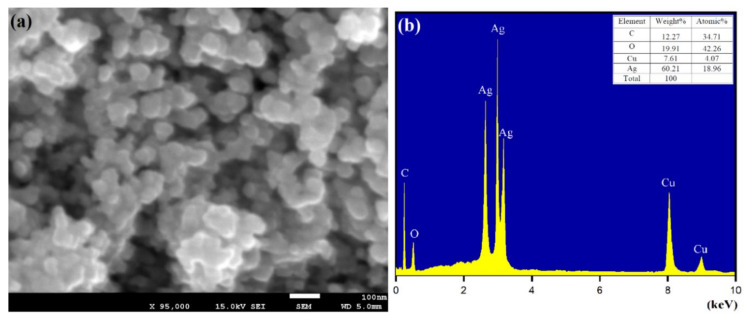
(**a**) SEM images and (**b**) EDX spectrum of biomolecule-capped AgNPs.

The scanning electron microscopy (SEM) and energy-dispersive X-ray spectroscopy (EDX) results accentuate the biomolecule-assisted synthesis of AgNPs, as shown in Figure 9a,b. The SEM analysis at 2 µm dimension scale shows the surface morphology of AgNPs with slight aggregation showing monodisperse spherical-shaped surface morphology. The EDX spectrum of AgNPs deposited on the copper grid conforms to the *Matricaria chamomilla* L. extract containing Cu, Ag, and O in an aggregation (existing in flowers and cylindrical appearance) as observed in SEM (Figure 9b). However, the presence of Cu and O is expected from the gird used to analyze the *Matricaria chamomilla* L. extract sample solution. The observed peaks at 2.9, 3.1, and 3.3 keV are the corresponding binding energies of Ag Lα, Ag Lβ, and Ag Lβ2. The obtained optical absorptions are the matching silver nanocrystalline peaks because of surface plasmon resonance [63,64]. Based on the obtained results, the biomolecule-assisted synthesis of AgNPs of *Matricaria chamomilla* L. extract was confirmed.

**Figure 10:**
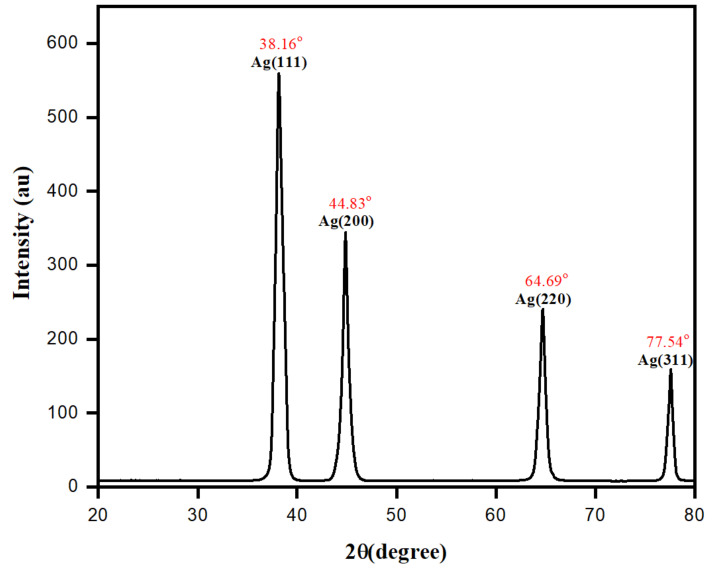
X-ray diffraction (XRD) pattern of biomolecule-assisted AgNPs.

The crystalline structure of the AgNPs was studied using XRD analysis, as shown in Figure 10. The crystalline powder for XRD analysis was acquired from the dark brown silver sol after centrifugation, followed by multiple washings and complete drying in an oven. At a wide angular range of 20° ≤ 2θ ≤ 80°, XRD data of biomolecule-capped AgNPs were collected and discussed. As shown in Figure 10, the crystalline existence and purity of as-prepared AgNPs were confirmed by studying the XRD pattern. The observed diffraction peaks at 2θ of 38.16, 44. 83, 64.79, and 75.54 were indexed to Bragg reflections (111), (200), (220), and (311), respectively. The XRD pattern was obtained as per the standard powder diffraction (JCPDS) file no: 04-0783 corresponding to the silver metallic nanoparticles of the crystalline face-centered cubic (fcc) planer geometry. However, a similar finding was observed for AgNPs with intense Bragg reflected planes of diffraction [65,66]. The intensity of the peak corresponding to the (111) plane was greater than that of other planes, promoting the idea that AgNPs predominantly established along the (111) direction. Analysis of the X-ray diffraction (XRD) showed that most of the AgNPs were spherical. The average estimated crystallite size of the AgNPs estimated from the FWHM of the diffraction peak using the Scherrer equation, *D* = *Kλ*/*β*cos*θ*, was found to be 12.02 nm. As illustrated above, these findings were roughly consistent with the sizes of nanoparticles derived from the TEM analysis.

**Figure 11:**
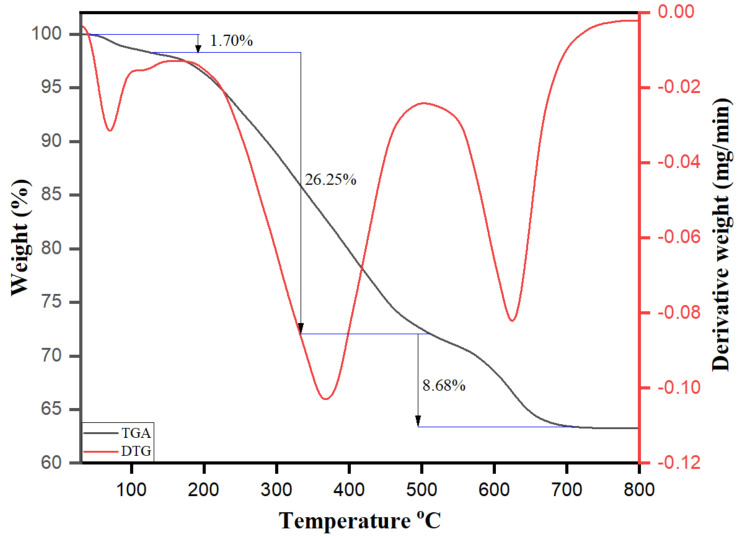
TGA/DTG thermogram of biomolecule-capped AgNPs.

The simultaneous thermal gravimetric analysis/derivative thermogravimetry analysis (TGA/DTG) was performed to validate the development of stable AgNPs and determine the thermal stability of AgNPs using *M. chamomilla* extract as a capping or stabilizing agent. The TGA and DTG analyses are primarily conducted ahead to elevate the thermal stability and the weight loss by thermal degradation patterns accomplished in the presence of capping or stabilizer agents onto the surface of AgNPs. The literature accentuates the efficacy of biological molecules’ effectiveness as reducing/capping agents to determine the composition, shape, size, and surface charge of the growing nanoparticles [32,67]. Figure 11 displays the TGA/DTG curve of AgNPs, as recorded at a heating rate of 10 °C/min between 30 and 800 °C with the sample under a nitrogen (N_2_) atmosphere. The thermal decomposition of the AgNPs occurs in several steps and shows a total weight loss of 36.63% after thermal analysis to 800 °C. The first weight loss of 1.70% from 30 to 130 °C is due to the moisture content and absorbed water molecules present on the surface of the AgNPs. Upon further heating, the sample exhibited a second weight loss of 26.25% between the temperature range of 130 and 510 °C and the final weight loss of 8.68% took place at a temperature range of 510 to 690 °C. The degradation was observed between 110 and 510 °C, with a related weight loss of approximately 26.25% because of the decomposition of organic biomolecules present on the surface of AgNPs. The decomposition of phenolic, flavonoid, and other biomolecules originating in the *Matricaria chamomilla* extract, which are responsible for stabilizing AgNPs, can be attributed to this weight loss. In addition to a steady weight loss of approximately 8.68% above 510 °C, a thermal degradation of resistant aromatic compounds on the surface of AgNPs will probably be attributed to the cause [68]. These results are attributed to the vital role of the biomolecules present in the *Matricaria chamomilla* extract in the nucleation, growth, and stabilization of AgNPs. The thermal stability of biomolecule-assisted AgNPs depends directly on the decomposition temperature of their different functional groups. No further weight loss was observed upon additional heating to 800 °C. 

### 3.6. Photocatalytic Dye Degradation of Rhodamine B

**Figure 12:**
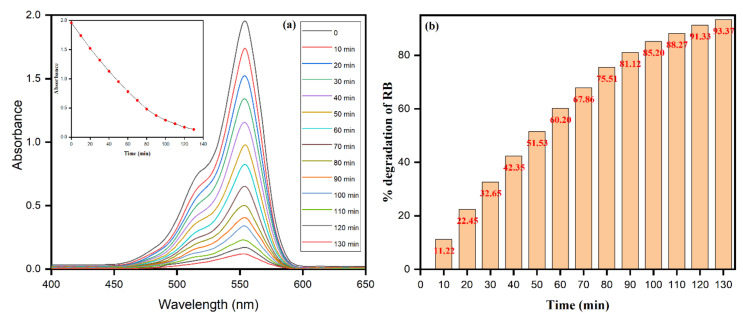
(**a**) Photocatalytic degradation of RB dye at different time intervals in the presence of AgNPs. (**b**) Effect of irradiation time on the photocatalytic degradation of RB by AgNPs efficiency.

The photocatalytic reduction reaction was intended for the degradation of staining fluorescent dye Rhodamine B (RB) using photo-induced biomolecule-assisted AgNPs as photocatalysts under UV light. The phenomenon of photocatalysis is after the competitive mutual recombination and the separation of electron–hole pairs [69]. However, an increase in the electron–hole pair separation in a photocatalytic reduction reaction leads to an increase in photocatalytic activity throughout the lifetime of charge carriers [70]. The study appraises the photocatalytic degradation of AgNPs against RB in the wavelength ranging from 400 to 650 nm. The intense characteristic peak at ca. 554 nm, as ascertained by the physical appearance of the pink aqueous solution of RB, was reduced (as observed from the colorless appearance) with an increase in time intervals. The close perusal of Figure 12a emphasizes the photodegradation of RB molecules in the presence of AgNPs. The results are accentuated from the decreased intensity of peak ca. 554 nm with increased time intervals, and the percent degradation of dye (RB) molecules increases with increased time intervals. After 130 min, the percent degradation of RB was observed as 93.37%, as depicted in Figure 12b.

### 3.7. Effect of Temperature

**Figure 13:**
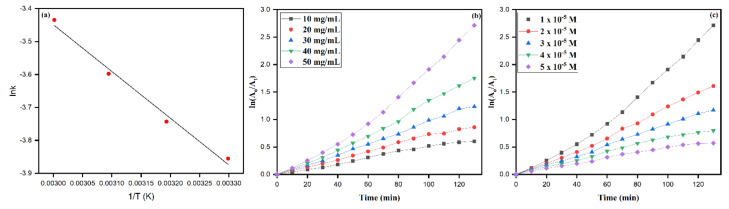
(**a**) Arrhenius plot (lnk vs. 1/T curve) for calculation of activation energy (Ea). (**b**) First-order photocatalytic degradation kinetics at different AgNPs catalyst dosages (plots of ln(A_o_/A_t_) vs. irradiation time (min)). (**c**) Plots of ln(A_o_/A_t_) vs. irradiation time (min) as a function of initial RB dye concentration.

The changing temperature directly affects the formation, shape, and size distribution of AgNPs [71]. As such, temperatures have a significant influence on the occurrence of chemical reactions. The temperature effect on RB degradation was optimized by continuous UV irradiation on the reaction mixture in a thermostatic chamber with the temperature ranging from 30 to 60 °C while keeping other parameters constant. The result obtained, as summarized in Table 1, emphasizes that the percent degradation of RB increases with an increase in the temperature, such as 30 (percent degradation 93.36) to 60 °C (percent degradation 98.55). The optimum temperature for RB’s catalytic degradation was 30 °C for our study with significant RB dye degradation. In Figure 13a, from the photodegradation of RB dye molecules in the presence of AgNPs, the lnk values were calculated (Equation (8)). The Arrhenius equation was utilized in the photodegradation reduction reaction as to determine the activation energy:

(8) $$\ln k=\;\frac{-Ea}{RT}+\ln A$$

where *k* is the rate constant, *E_a_* is the activation energy (J/mol), *R* is the gas constant (8.314 J·mol^−1^·K^−1^), *A* is the pre-exponential factor, and *T* is the temperature in Kelvin. Meanwhile, the activation energy was obtained from the *lnk* vs. *1/T* curve and was calculated to be 11.79 KJ/mol. The first-order kinetics was applied to deduce the RB degradation over AgNPs as catalytic doses, extending from 10 to 50 mg/ml, as depicted in Figure 13b.

### 3.8. Effect of pH

The reaction mixture’s initial pH influences the photocatalytic degradation’s properties behind the active species formation (radicles) [72]. The reaction media, whether alkaline or acidic, are inferred from pH values and come after the formation of hydroxy radicles. Thus, the study pertains to deducing the influence of pH on RB’s photodegradation in a wide initial pH range from pH 2 to pH 8. The reaction mixture’s initial pH was adjusted by adding 0.1 (M) HCl or NaOH; the pH was adjusted from acidic to alkaline salinity, as summarized in Table 1. The results emphasize the increase in both the temperature and the pH values. The optimum degradation was attained at a comparatively higher temperature and alkaline medium. At relatively high temperatures, i.e., 60 °C and at an alkaline pH 8, the percent degradation of RB was found to be higher such as 98.55% and 93.36% in comparison to other lower temperatures and numerical pH values, respectively (Table 1). In an alkaline pH, a mixture solution accelerates the generation of **^·^**OH radicles, leading to an increase in RB molecules’ rate of degradation with maximum degradation efficacy. Similar results have been observed in RB’s photodegradation using ZnO nanopowder and in the photodegradation of RB using AgNPs from *Shorea robusta* leaf extract [73,74]. 

### 3.9. Role of Catalyst Dosage and Initial RB Dye Concentration 

The increases in the catalytic concentration are after the improved photodegradation of dye molecules. The phenomenon was believed to be behind the possibilities of an improved adsorbed number of photons and availabilities of increased reaction active sites onto the catalyst’s surface with an increase in concentrations. The observations of increased concentrations with an increased degradation rate of RB are summarized in Table 2. The linear increase in the rate constant (*k*) values from 0.00201 to 0.02117 was observed with increased catalytic concentrations (AgNPs). As observed, the results are found with an increase in the catalytic dose, and more active sites are available for the degradation of dye (RB) molecules. Besides, the initial concentration from 1 × 10^−5^ to 5 × 10^−5^ mol^−1^ of dye (RB), keeping other parameters constant, was comprehended, as shown in Figure 13c. The observed decreased trends of the rate constants with increased initial dye concentrations were observed after reaching the limited number of dye molecules onto the nanocatalyst’s surface to degrade. However, the rate of the reaction was determined as per the equation (Equation (9))

(9) $$\ln(A)=kt+\ln(A_{0})$$

The rate of reactions was calculated by using the above Equation (5) and for the concentrations of 1.0 ×10^−5^, 2.0 ×10^−5^, 3.0 ×10^−5^, 4.0 ×10^−5^, and 5.0 ×10^−5^, the rate constants *k* were 0.02117, 0.01275, 0.00927, 0.00641, and 0.00462, respectively. Since the catalytic concentration was constant while increasing the initial dye concentrations, the possibility of available active sites on the nanocatalyst surface decreases; thus, this leads to the decrease in the rate constants with an increase in initial dye concentrations and an increase in time intervals, and the competition between reaction intermediates, while dye molecules fasten to occupy the available active sites on the surface of the catalyst. The inhibition effects that played their role in the higher concentration of RB to be adsorbed onto the catalytic surface result in decreased direct contact of dye molecules and, thus, the observed decreased rates of reaction *k* numerical values, as shown in Table 2. 

### 3.10. The Plausible Mechanism behind the Photodegradation of RB Dye 

**Figure 14:**
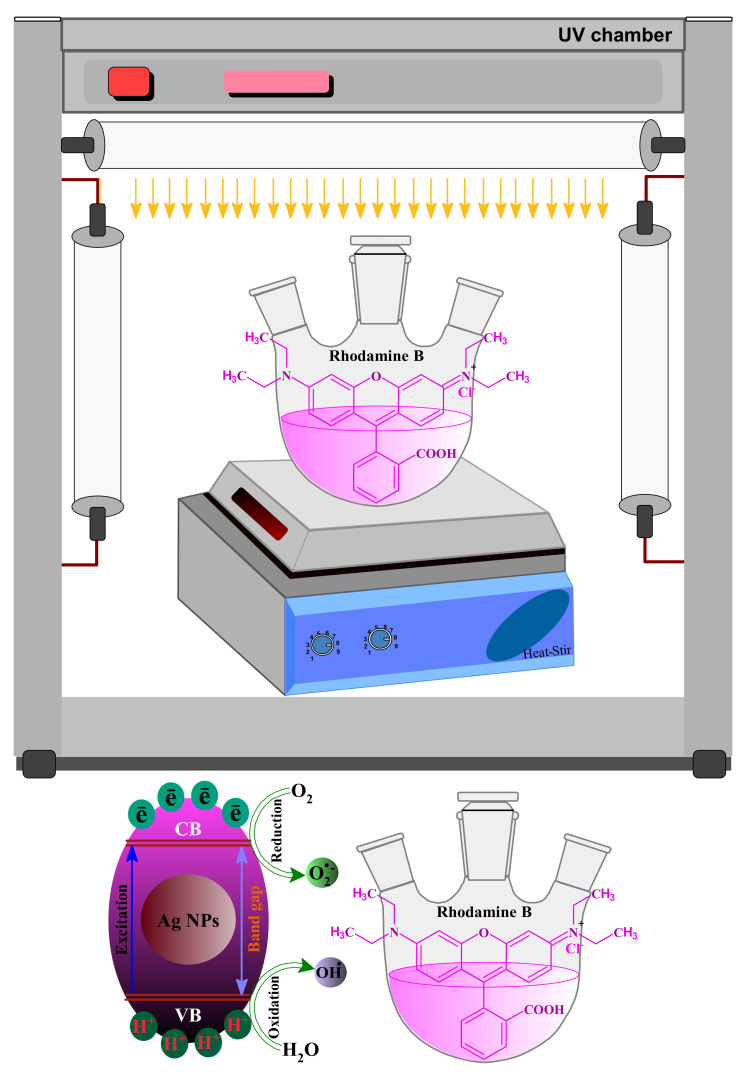
Schematic diagram showing the plausible mechanism for the degradation of RB over biomolecule-capped AgNPs under UV light irradiation.

The catalytic photodegradation was assumed to have functioned on the surface of the nanocatalysts (AgNPs). In the presence of UV light irradiation, the electrons from the valence band (VB) are excited to the higher-energy conduction band (CB) in the ongoing process as positive (h^+^ VB), and conduction electrons (ē CB) are generated. The rise of such photo-generated species (h^+^ VB and ē of CB) is responsible for the photodegradation upon the reduction reaction of RB molecules in a reaction mixture with the formation of highly reactive radicles. The h^+^ VB in a reaction mixture leads to the formation of hydroxyl radicles (**﮲**OH) by reaction with water molecules; meanwhile, the dissolved oxygen (O_2_) is converted to superoxide radicle anions (O_2_**⁻**^•^) when reacting with e^−^ C_B_ by AgNPs [69], as depicted in the systematic Figure 14. The photo-generated O_2_**⁻**^•^ can react with the reaction mixture’s water molecules to finally emerge with **﮲**OH and hydroperoxyl radicles (HO_2_**﮲**). The photo-generated O_2_**⁻**^•^, **﮲**OH, and HO_2_^•^ radicles are believed to have their worth in their appealing role in the photocatalytic degradation of RB molecules [75]. Moreover, in the presence of these radicles, the RB molecules degraded to fewer toxic fragments, including ammonium (NH_4_^+^), carbon dioxide (CO_2_), nitrate (NO_3_^−^), and water molecules in the presence of AgNPs. 

### 3.11. Stability and Reusability of the Phytochemical-Assisted AgNPs as a Catalyst 

**Figure 15:**
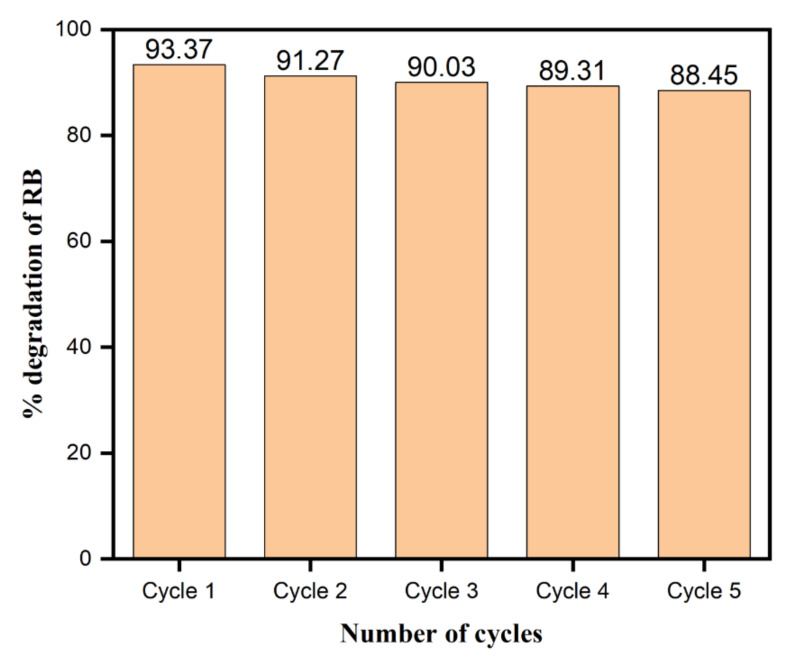
Recyclability study of AgNPs as a photocatalyst for the photodegradation of RB dye.

In Figure 15, the percent degradation of RB in the presence of AgNPs was comprehended with several cycles to deduce the stability and reusability of AgNPs as a nanocatalyst. In the photocatalytic degradation stability, the functional applicability and recyclability of nanoparticles possess appealing importance. After each cycle, keeping the recyclability and nanocatalyst’s stability in consideration, the use of AgNPs as a catalyst was estimated for the RB degradation under visible light. The samples were centrifuged to isolate the catalyst from the mixed solution and were washed with deionized water and dried for about 1 hour at 100 °C. The nanoparticles gained their original physical appearance after washing and drying and, as such, were used for the next cycle treatment and so on. As depicted in Figure 15, showing the results of the photocatalytic performance of the nanocatalysts, the slight decrease in the dye degradation with consecutive cycles was expected from the minor loss and deactivation of the catalyst in the cycling experiment. However, the overall performance of AgNPs as a nanocatalyst makes them worthy to be applied and suitable, with higher stability and durability.

Numerous experiments use various nanoparticles as catalysts for the photocatalytic degradation of RB dye under different light sources. The current research utilizes the biomolecule-capped AgNPs for RB degradation under UV light irradiation. Earlier studies on RB degradation using UV light, visible light, and sunlight in the presence of different catalysts are compiled and compared with the present work in Table 3. Compared to all other approaches, the catalyst synthesis process used in the present study is a straightforward and inexpensive one with excellent photocatalytic efficiency.

## 4. Conclusions

The study provides an eco-friendly photo-induced biomolecule-assisted synthesis of AgNPs using *Matricaria chamomilla* L. extract. The optimization was carried out to maximize the yield of AgNPs within 45 min bright sunlight exposure without any external means of energy or instrumental support such as heating or stirring. The biomolecule-assisted synthesis of AgNPs was optimized through the concentration of AgNO_3_ in the reaction mixture upon time intervals between 5 and 45 min in bright sunlight. The concentration of 4% (*v*/*v*) *Matricaria chamomilla* L. extract at 45 min was obtained as the optimum concentration of AgNO_3_ used for the controlled biomolecule-assisted synthesis of AgNPs. The biomolecule-capped AgNPs were in different sizes and shapes, but most were spherical with an average size of about 26 nm. From the facts of the obtained results, the probable mechanism after the biomolecule-assisted synthesis of AgNPs was proposed and explained. The photocatalytic degradation of RB was determined to be affected by different kinetic parameters, including nature and dye concentration, AgNPs concentration, pH, and dye solution temperature. Moreover, it was demonstrated that the AgNPs that show significant catalytic properties for RB dye degradation are stable enough to be recycled several times for reusability. The as-prepared AgNPs can be a promising material for wastewater treatment under UV light. 

## Figures and Tables

**Table 1:** Effect of temperature and pH on the degradation efficiency (%) and rate constant.

Temperature ºC	% Degradation	Rate Constant (k)	R^2^	pH	% Degradation	Rate Constant (k)	R^2^
30	93.36	0.02117	0.982	2	70.85	0.00972	0.968
40	95.25	0.02368	0.982	4	80.23	0.01259	0.978
50	97.12	0.02736	0.975	6	87.74	0.01663	0.981
60	98.55	0.03225	0.967	8	93.36	0.02117	0.982

**Table 2:** Effect of RB and AgNPs dosages on the degradation efficiency (%) and rate constant.

RB Dye. (M)	% Degradation	Rate Constant (k)	R^2^	AgNPs (mg)	% Degradation	Rate Constant (k)	R^2^
1.0 × 10^−5^	93.36	0.02117	0.982	10	45.40	0.00201	0.989
2.0 × 10^−5^	79.59	0.01275	0.995	20	57.65	0.00692	0.989
3.0 × 10^−5^	67.85	0.00927	0.997	30	70.91	0.00987	0.997
4.0 × 10^−5^	54.55	0.00641	0.990	40	82.65	0.01386	0.993
5.0 × 10^−5^	39.79	0.00462	0.989	50	93.36	0.02117	0.982

**Table 3:** Comparative study of the photocatalytic efficiencies of the different photocatalysts.

Catalyst	Light Source	Irradiation Time	Degradation Efficiency (%)	Ref.
Au-ZnO	UV light	180 min	95%	[76]
ZnO	Sunlight	200 min	98%	[77]
ZnO	UV light	70 min	97.75%	[78]
Zeo-TiO_2_	UV irradiation	80 min	100%	[79]
Zeo-ZnO	UV irradiation	80 min	81%	[79]
TiO_2_ film	UV irradiation	30 min	98.46%	[80]
ZnO/Ag	Visible light	120 min	99%	[81]
AgNPs	UV irradiation	100 min	90.41%	[74]
AgNPs	UV irradiation	130 min	93.39%	Present Work
